# Sustained induction of IP-10 by MRP8/14 via the IFNβ–IRF7 axis in macrophages exaggerates lung injury in endotoxemic mice

**DOI:** 10.1093/burnst/tkad006

**Published:** 2023-09-11

**Authors:** Juan Wang, Guiming Chen, Lei Li, Sidan Luo, Bingrong Hu, Jia Xu, Haihua Luo, Shan Li, Yong Jiang

**Affiliations:** Guangdong Provincial Key Laboratory of Proteomics, State Key Laboratory of Organ Failure Research, Department of Pathophysiology, School of Basic Medical Sciences, Southern Medical University, Guangzhou 510515, Guangdong, China; Guangdong Provincial Key Laboratory of Proteomics, State Key Laboratory of Organ Failure Research, Department of Pathophysiology, School of Basic Medical Sciences, Southern Medical University, Guangzhou 510515, Guangdong, China; Guangdong Provincial Key Laboratory of Proteomics, State Key Laboratory of Organ Failure Research, Department of Pathophysiology, School of Basic Medical Sciences, Southern Medical University, Guangzhou 510515, Guangdong, China; Guangdong Provincial Key Laboratory of Proteomics, State Key Laboratory of Organ Failure Research, Department of Pathophysiology, School of Basic Medical Sciences, Southern Medical University, Guangzhou 510515, Guangdong, China; Guangdong Provincial Key Laboratory of Proteomics, State Key Laboratory of Organ Failure Research, Department of Pathophysiology, School of Basic Medical Sciences, Southern Medical University, Guangzhou 510515, Guangdong, China; Guangdong Provincial Key Laboratory of Proteomics, State Key Laboratory of Organ Failure Research, Department of Pathophysiology, School of Basic Medical Sciences, Southern Medical University, Guangzhou 510515, Guangdong, China; Guangdong Provincial Key Laboratory of Proteomics, State Key Laboratory of Organ Failure Research, Department of Pathophysiology, School of Basic Medical Sciences, Southern Medical University, Guangzhou 510515, Guangdong, China; Guangdong Provincial Key Laboratory of Proteomics, State Key Laboratory of Organ Failure Research, Department of Pathophysiology, School of Basic Medical Sciences, Southern Medical University, Guangzhou 510515, Guangdong, China; Guangdong Provincial Key Laboratory of Proteomics, State Key Laboratory of Organ Failure Research, Department of Pathophysiology, School of Basic Medical Sciences, Southern Medical University, Guangzhou 510515, Guangdong, China

**Keywords:** Endotoxemia, Interferon-inducible protein 10, Interferon beta, Macrophage, Myeloid-related protein 8/14, Interferon regulatory factor-7

## Abstract

**Background:**

As a damage-associated molecular pattern, the myeloid-related protein 8/14 (MRP8/14) heterodimer mediates various inflammatory diseases, such as sepsis. However, how MRP8/14 promotes lung injury by regulating the inflammatory response during endotoxemia remains largely unknown. This study aims at illuminating the pathological functions of MRP8/14 in endotoxemia.

**Methods:**

An endotoxemic model was prepared with wild-type and myeloid cell-specific *Mrp8* deletion (*Mrp8*^ΔMC^) mice for evaluating plasma cytokine levels. Lung injury was evaluated by hematoxylin and eosin (H&E) staining, injury scoring and wet-to-dry weight (W/D) ratio. The dynamic profile of interferon γ (IFNγ)-inducible protein 10 (IP-10) mRNA expression induced by macrophage MRP8/14 was determined by quantitative real-time polymerase chain reaction (qPCR). Immunoblotting was used to evaluate the increase in IP-10 level induced by activation of the JAK–STAT signaling pathway. Luciferase reporter assay was performed to detect the involvement of IRF7 in *Ip-10* gene transcription. *In vivo* air pouch experiments were performed to determine the biological function of IP-10 induced by MRP8/14.

**Results:**

Experiments with *Mrp8*^ΔMC^ mice showed that MRP8/14 promoted the production of cytokines, including IP-10, in the bronchoalveolar lavage fluid (BALF) and lung injury in endotoxic mice. The result of qPCR showed sustained expression of *Ip-10* mRNA in macrophages after treatment with MRP8/14 for 12 h. Neutralization experiments showed that the MRP8/14-induced *Ip-10* expression in RAW264.7 cells was mediated by extracellular IFNβ. Western blotting with phosphorylation-specific antibodies showed that the JAK1/TYK2-STAT1 signaling pathway was activated in MRP8/14-treated RAW264.7 cells, leading to the upregulation of *Ip-10* gene expression. IRF7 was further identified as a downstream regulator of the JAK–STAT pathway that mediated *Ip-10* gene expression in macrophages treated with MRP8/14. *In vivo* air pouch experiments confirmed that the IFNβ-JAK1/TYK2-STAT1-IRF7 pathway was required for chemokine (C-X-C motif) receptor 3 (CXCR3)^+^ T lymphocyte migration, which promoted lung injury in the context of endotoxemia.

**Conclusions:**

In summary, our study demonstrates that MRP8/14 induces sustained production of IP-10 via the IFNβ-JAK1/TYK2-STAT1-IRF7 pathway to attract CXCR3^+^ T lymphocytes into lung tissues and ultimately results in lung injury by an excessive inflammatory response in the context of endotoxemia.

## Highlights

- MRP8/14 promotes sustained induction of IP-10 in lung tissues and is confirmed to be involved in lung injury of endotoxic mice.
- IP-10 induction by MRP8/14 was mediated by sequential activation of the IFNβ-JAK1/TYK2-STAT1-IRF7 signaling pathway.
- Prolonged production of IP-10 attracts CXCR3^+^ lymphocytes to lung tissues and triggers an exaggerated inflammatory response and lung injury during endotoxemia via the IFNβ–IRF7 axis.

## Background

Sepsis is a life-threating organ dysfunction due to infection, and lung injury is one of the severe complications, which is characterized by an excessive inflammatory response in the lung [1,2]. The mortality rate of septic patients varies from 34 to 82%, depending on the severity of lung injury [1]. Previous studies have shown that damage-associated molecular patterns are closely related to the occurrence and progression of sepsis-induced lung injury [3,4].

Myeloid-related protein (MRP) 8 and MRP14, which are also known as S100A8 and S100A9, respectively, exist in a heterodimeric form (MRP8/14) [5,6]. MRP8 and MRP14 account for almost half of the soluble cytosolic proteins in neutrophils and are also abundant in monocytes, macrophages and epithelial cells [5,7,8]. MRP8/14 is released into the extracellular space from cells in response to various stimuli and acts as a functional ligand for Toll-like receptor 4 (TLR4) [9] and receptor for advanced glycation end products [10]. MRP8/14 was reported to participate in multiple cellular processes, including proliferation, differentiation, chemotaxis, migration and cytoskeletal rearrangement [11,12]. High levels of MRP8/14 have been detected in many inflammatory and immune-related diseases, such as sepsis, inflammatory bowel disease, rheumatoid arthritis and myocardial infarction [9,13–15]. A previous study demonstrated that MRP8/14 enhanced or prolonged inflammatory reactions by inducing tumor necrosis factor α (TNF-α) release [9] and caused cell death [16]. Recently, the role of MRP8/14 in the pathogenesis of lung injury has gained increasing attention.

Our previous study showed that MRP8/14 immediately induced interferon γ (IFNγ) inducible protein 10 (IP-10) expression in monocytes/macrophages [17]. We further identified that early-phase IP-10 induction was mediated by the TLR4-TRIF-NF-κB/IRF3 signaling pathway in monocytes/macrophages treated with MRP8/14 [17]. As a CXC chemokine, IP-10 can specifically bind to chemokine (C-X-C motif) receptor 3 (CXCR3) to promote T lymphocyte migration and neutrophil infiltration [18–20], and the IP-10–CXCR3 axis has been suggested to be critical for exacerbating the pathological process of sepsis [21,22].

In the present study, we found that the sustained expression of *Ip-10* mRNA in macrophages was exhibited as double peaks in response to treatment with MRP8/14. Based on this finding, we examined the function and mechanism of MRP8/14-induced late-phase IP-10 production in the context of endotoxemia and showed that MRP8/14 promoted inflammation and lung injury in the context of endotoxemia through the IFNβ-JAK1/TYK2-STAT1-IRF7-IP-10-CXCR3 signaling pathway.

## Methods

### Mice and reagents

Male C57BL/6 wild-type (WT), *Mrp8*^ΔMC^, *Irf7*^−/−^ and *Cxcr3*^−/−^ mice were purchased from Cyagen Biosciences Inc. (Taicang, China). RAW264.7 cells were purchased from the American Type Culture Collection (ATCC, Manassas, VA, USA). Lipopolysaccharide (LPS) (serotype O111B4) and a Limulus amebocyte lysate kit were purchased from Sigma-Aldrich (St. Louis, MO, USA). Detoxi-Gel™ endotoxin removing gel was obtained from Pierce (Rockford, IL, USA). Mouse IP-10, interleukin (IL)-1β, IL-6, TNF-α and monocyte chemoattractant protein-1 (MCP-1) immunoassay kits and polyvinylidene difluoride membranes were obtained from Millipore (Billerica, MA, USA). Enzyme-linked immunosorbent assay (ELISA) kits for mouse MRP8/14, IFNα and IFNβ were purchased from Sabbiotech (College Park, MD, USA), Invitrogen (Carlsbad, CA, USA) and PBL Assay Science (Piscataway, NJ, USA), respectively. Mouse IFNβ (mIFNβ) and IFNβ neutralizing antibody (Ab) were purchased from PBL Assay Science (Piscataway, NJ, USA) and IP-10 neutralizing Ab was purchased from R&D Systems (Minneapolis, MN, USA). All specific Abs against phospho-JAK1, JAK2, JAK3, TYK2 and STAT1 were purchased from Cell Signaling Technology (Danvers, MA, USA). Abs against mouse JAK1, JAK2, JAK3, TYK2 and STAT1 were purchased from Santa Cruz Biotechnology (Santa Cruz, CA, USA). Abs against mouse IRF7 and Lamin B1 were obtained from Abcam (Cambridge, MA, USA). GLPG0634 and fludarabine were purchased from Selleck (Houston, TX, USA). The ReverTra Ace-α-™ kit was obtained from Toyobo (Osaka, Japan). The Fast Start Universal SYBR Green Master Kit was purchased from Roche (Mannheim, Germany). NE-PER™ nuclear and cytoplasmic extraction reagents and Super Signal West Pico enhanced chemiluminescence reagents were obtained from Thermo Fisher Scientific (Waltham, MA, USA). The Dual-Glo luciferase assay system was purchased from Promega (Madison, WI, USA). Lipofectamine 3000 was obtained from Invitrogen (Carlsbad, CA, USA). The mouse anti-CXCR3 Alexa Fluor 488-conjugated Ab was obtained from R&D Systems (Minneapolis, MN, USA).

### Animal model

Six- to eight-week-old male C57BL/6 mice were purchased from the Animal Center of Southern Medical University (Guangzhou, China) and housed under specific pathogen-free conditions with a 12-h light/dark cycle and free access to food and water. All animal experiments in this study were performed in accordance with the National Institutes of Health guidelines and were approved by the Bioethics Committee of Southern Medical University (Guangzhou, China).

To establish the endotoxemia model, WT C57BL/6 mice were intraperitoneally (i.p.) injected with LPS (20 mg/kg). Equal volume of normal saline (NS) was used as control. WT mice were subjected to cecum ligation and puncture, as previously described [23]. Briefly, anesthetized mice were subjected to midline laparotomy in a sterile field. The cecum was identified and was ligated and punctured once with a 21-gauge hypodermic needle followed by slight compression to release a small amount of intestinal contents. Then, the cecum was returned to the peritoneal cavity and the abdominal skin was closed. In parallel, the mice in the sham group underwent the same procedure without ligation or puncture. The animals were resuscitated by subcutaneous administration of 1 ml of normal saline immediately after surgery.

To clarify the role of MRP8/14 in lung injury, WT C57BL/6 mice were intravenously (i.v.) injected with MRP8/14 heterodimers (4 mg/kg), denatured MRP8/14 (dMRP8/14) (4 mg/kg) or enhanced green fluorescent protein (EGFP) (4 mg/kg). To examine whether MRP8/14 can rescue the LPS-induced response in *Mrp8* gene-deficient mice, *Mrp8*^ΔMC^ mice were injected with LPS (20 mg/kg, i.p.) and MRP8/14 heterodimers (4 mg/kg, i.v.) 1 h after LPS administration. For the IP-10 neutralization experiment, WT or *Mrp8*^ΔMC^ mice were pretreated with the neutralizing IP-10 Ab (2 mg/kg, i.v.) or control IgG (2 mg/kg, i.v.) 2 h before being administered LPS or MRP8/14. The mice were sacrificed, and blood and tissue collection were performed 12 h after modeling unless otherwise noted.

An *in vivo* air pouch mouse model was established as previously described [24–26]. In brief, after the mice were anesthetized, 5 ml of sterile air was subcutaneously injected into the intrascapular area of the back. An additional 3 ml of sterile air was injected into the air cavity again on day 3. On day 5, the mice were i.p. injected with LPS to activate lymphocytes with high CXCR3 expression. Supernatants of bone marrow-derived macrophages (BMDMs) from WT or *Irf7*^−/−^ mice were stimulated with or without MRP8/14 for 36 h and then injected into the dorsal air pouches of WT mice after LPS stimulation for 8 h. Then, precooled sterile PBS was injected into the air pouches and the exudates were collected. The number of migrated leukocytes in the exudates was counted using Wright–Giemsa staining and a Coulter counter, and the proportion of CXCR3^+^ T lymphocytes among the migrated cells was determined with mouse anti-CXCR3 Alexa Fluor 488-conjugated Ab by flow cytometry (FACSVerse, BD Biosciences, San Jose, CA, USA).

### MRP8/14 heterodimer preparation

Mouse MRP8/14 heterodimer and EGFP were prepared as described [17,27]. Endotoxin contaminants were removed using the Detoxi-Gel™ endotoxin removing gel, and the final endotoxin contaminants in the MRP8/14 heterodimer or EGFP preparations were confirmed by a Limulus amebocyte lysate assay (minimum LPS sensitivity = 0.125 EU/ml). To obtain denatured MRP8/14, the MRP8/14 heterodimer was heated at 80°C for 30 min [9].

### Cell culture and alveolar macrophage collection

RAW264.7 cells were cultured in Dulbecco’s modified Eagle’s medium supplemented with 10% fetal bovine serum, 100 U/ml penicillin and 100 mg/ml streptomycin at 37°C in a 5% CO_2_ incubator. BMDMs were harvested and grown in Dulbecco’s modified Eagle’s medium containing 10% fetal bovine serum, 100 U/ml penicillin, 100 mg/ml streptomycin and macrophage colony-stimulating factor (10 ng/ml) for 7 days [28]. After being pretreated with the IFNβ neutralizing Ab (1 μg/ml), GLPG0634 (500 nM) or fludarabine (10 μM) for 1 h, the cells were stimulated with MRP8/14 (1.5 μg/ml) for different times. Alveolar macrophages (AMs) were isolated from the bronchoalveolar lavage fluid (BALF) of mice as previously described [29].

### Cytokine quantitation

Plasma IL-1β, TNF-α, IL-6, MCP-1 from mice, IP-10 from BALF of mice or cultured cell supernatants were measured using Luminex multiplex immunoassay bead array technology [23,30,31]. IFNα and IFNβ levels from cultured cell supernatants were quantified by ELISA, according to the manufacturer’s instructions.

### Quantitative real-time polymerase chain reaction

Total RNA was extracted from cultured or isolated primary cells using TRIzol reagent in accordance with the manufacturer’s procedures. After reverse transcription, quantitative real-time polymerase chain reaction (qPCR) amplification was performed with the resulting cDNAs on an ABI 7500 (Applied Biosystems, Foster City, CA, USA) using a standard protocol for 40 cycles. Gene transcripts were quantified and normalized to beta actin (*Actb*) mRNA. The primers used for qPCR are listed in supplementary Table S1 (see online supplementary material).

### Protein extraction from lung tissues and cultured cells

For cellular total protein extraction, the lung tissues (~5 mg) were crushed in a mortar and pestle cooled with liquid nitrogen and then placed in precooled 1.5 ml microcentrifuge tubes. RIPA buffer (~300 μl) (Thermo Fisher Scientific, Waltham, MA, USA) containing a cocktail of protease inhibitors and phenylmethanesulfonyl fluoride was added to the samples and sonicated four times for 3 s at 25% power with 9 s intervals using a VCX800 ultrasonic cell homogenizer (Sonics, Newtown, CT, USA). RAW264.7 cells were collected by centrifugation at 500 × g for 5 min at 4°C, followed by resuspension in 300 μl of lysis buffer and sonication as described above. Then, all samples were centrifuged at 13000 rpm for 25 min at 4°C and the supernatant was collected. Nuclear proteins were extracted from cultured cells with NE-PER**™** nuclear and cytoplasmic extraction reagents according to the manufacturer’s procedures. The protein concentration was determined by using the bicinchoninic acid method.

### Western blotting

Samples containing equal amounts (50 μg) of proteins were separated by sodium dodecyl sulfate -polyacrylamide gel electrophoresis in 8–12% acrylamide gels and transferred to polyvinylidene difluoride membranes using a western system from Bio-Rad (Hercules, CA, USA). The membranes were immediately placed in blocking buffer containing 5% bovine serum albumin (BSA) at room temperature for 1 h. The membranes were then incubated with specific primary Abs at 4°C overnight. After three washes for 5 min with Tris-buffered saline containing 0.1% Tween-20 washing buffer, the membrane was incubated with horseradish peroxidase-conjugated anti-mouse or rabbit secondary Abs at room temperature for 1 h. Enhanced chemiluminescence was used to visualize the protein bands and images were acquired using a ChemiDoc™ imaging system (Bio-Rad, Hercules, CA, USA).

### Dual luciferase reporter assay

The *Ip-10* promoter sequence was cloned into a pGL3 luciferase reporter vector (Promega, Madison, WI, USA) to construct a pGL3 luciferase reporter plasmid containing the *Ip-10* promoter (pGL3-*Ip10p*). The full sequence of *Irf7* was cloned into the pcDNA3 vector (pcDNA3-*Irf7*) to overexpress *Irf7* in RAW264.7 cells. RAW264.7 cells were cotransfected with the pGL3-*Ip10p* and pcDNA3-*Irf7* plasmids using Lipofectamine 3000 for 24 h. Then, the cells were stimulated with MRP8/14 for 12 h and lysed in passive lysis buffer. The extracts were centrifuged at 7500 × *g* for 1 min to remove cellular debris. A dual-luciferase assay system from Promega (Madison, WI, USA) was used to measure the activities of firefly and *Renilla* luciferases in the cell lysates. Relative luciferase activity was normalized to Renilla luciferase activity.

### Histopathological examination

After being fixed in 4% paraformaldehyde (Biosharp, ^#^BL539A), lung tissues were embedded in paraffin and cut into 5-μm-thick sections. The slices were stained with hematoxylin and eosin (H&E) for microscopic examination. The lung injury score in each field was quantified according to previous reports [32,33].

### Wet-to-dry lung weight ratio

Fresh lungs were harvested from the mice, weighed to determine the wet lung weight, placed in an oven at 80°C for 72 h and weighed again to determine the dry lung weight. The wet-to-dry lung weight ratio (W/D ratio) was calculated by dividing the wet weight by the dry weight.

### Statistical analysis

All data are presented as the mean ± SD and were analysed using SPSS v24.0 (IBM Corp, Armonk, NY, USA). Significant differences between groups that passed both normality (Shapiro–Wilk test) and equal variance (Levene test) tests were evaluated using one-way analysis of variance (ANOVA) followed by the Bonferroni *post hoc* test for multiple comparisons or the unpaired 2-tailed Student t test for comparison between two groups. If the data sets failed to show equal variance, Welch correction was performed, followed by Dunnett’s T3 multiple comparison exact probability test. A two-tail Mann–Whitney U test was used when the normality test failed between two groups. A value of *p* < 0.05 was considered statistically significant.

## Results

### MRP8/14 promotes cytokine production and lung injury in endotoxic mice

To clarify the relevance of MRP8/14 to endotoxemia, sera of LPS-induced septic shock or cecum ligation and puncture mice were collected for the detection of MRP8/14 protein expression. The results (supplementary Figure S1, see online supplementary material) showed that the levels of MRP8/14 were significantly increased in the sera from septic mice induced by LPS (supplementary Figure S1a) and cecum ligation and puncture modeling (supplementary Figure S1b).

**Figure 1:**
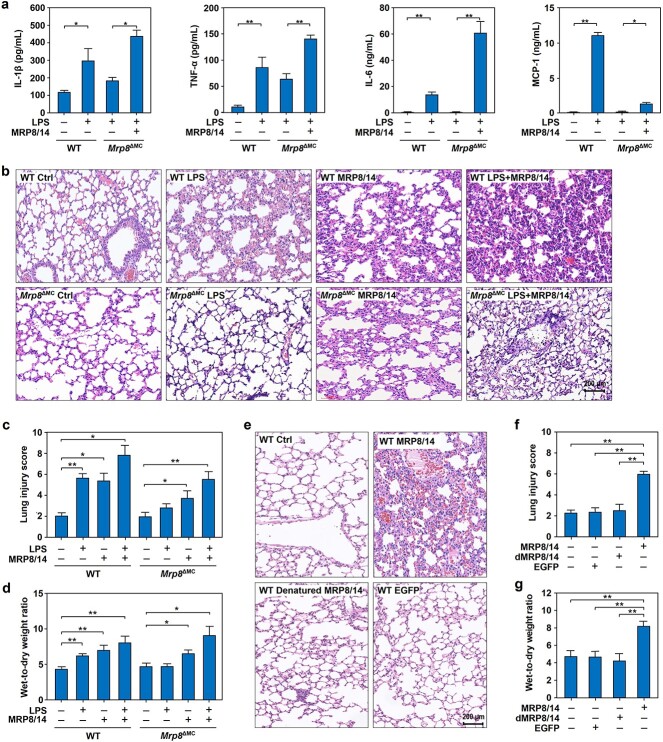
Effect of MRP8/14 on cytokine production and lung injury in endotoxic mice. (**a**) *Mrp8* gene deficiency reduces plasma proinflammatory cytokine levels in endotoxic mice. WT mice were injected intraperitoneally (i.p.) with normal saline (NS) or LPS (20 mg/kg). In *Mrp8* gene-deficient (*Mrp8*^ΔMC^) mice, MRP8/14 (4 mg/kg) or an equal volume of NS was intravenously (i.v.) injected after intraperitoneal administration of LPS (20 mg/kg) for 1 h. At 12 h after LPS administration, blood was collected, and plasma IL-1β, TNF-α, IL-6 and MCP-1 levels were measured by multiplex cytokine assays with a Luminex system. (**b**–**d**) *Mrp8* gene deficiency alleviates lung injury of endotoxic mice. WT or *Mrp8*^ΔMC^ mice were treated with NS, LPS (20 mg/kg), MRP8/14 (4 mg/kg) or LPS plus MRP8/14 for 12 h, followed by harvesting lung tissues from the mice and H&E staining. Representative histopathological images of lung tissues from WT mice and *Mrp8*^ΔMC^ mice are shown with a scale bar of 200 μm (b). Lung injury scores were determined by semiquantitative lung injury analysis (c). The W/D ratios of lung tissues in the different groups were calculated (d). (**e**–**g**) Effect of MRP8/14 injection on lung injury in mice. WT mice were injected with NS, MRP8/14 (4 mg/kg), equal amounts of denatured MRP8/14 (dMRP8/14) or EGFP (4 mg/kg), and lung tissues were collected 12 h after intravenous injection of MRP8/14. Histopathological examination was performed by H&E staining and representative histopathological images of lung tissues from WT mice are shown with a scale bar of 200 μm (e). Lung injury scores were calculated as described above (f). W/D ratios of the lung tissues of mice in the different groups were calculated (g). The data are expressed as mean ± SD and represent three independent experiments (*n* = 3). ^*^p < 0.05, ^*^^*^p < 0.01. *WT* wild-type, *LPS* lipopolysaccharide, *MRP8/14* myeloid-related protein 8/14, *IL-1β* interleukin-1β, *TNF-α* tumor necrosis factor α, *IL-6* interleukin-6, *MCP-1* monocyte chemoattractant protein-1, *W/D* wet-to-dry, *EGFP* enhanced green fluorescent protein, *H&E* hematoxylin and eosin

To determine the contribution of MRP8/14 to endotoxemia, WT and myeloid cell-specific *Mrp8-*knockout (*Mrp8*^ΔMC^) mice (supplementary Figure S2) were used to establish an endotoxic shock model by intraperitoneal injection of LPS. We found that the plasma levels of IL-1β, TNF-α, IL-6 and MCP-1 were dramatically increased in WT mice challenged with LPS, while *Mrp8* gene deficiency significantly blocked the induction of these cytokines by LPS, and this effect could be reversed by supplementation with MRP8/14 (Figure 1a).

We next sought to determine the effect of MRP8/14 on lung injury in the context of endotoxemia. WT and *Mrp8*^ΔMC^ mice were challenged with LPS, MRP8/14 or LPS plus MRP8/14. Histopathological examination results (Figure 1b) showed that the lung injury score (Figure 1c) and lung W/D ratio (Figure 1d) were significantly elevated in WT mice after LPS administration or MRP8/14 challenge. Lung injury score and W/D ratio were even higher in the mice treated with LPS plus MRP8/14 compared with LPS or MRP8/14 treatment alone. While *Mrp8* gene deficiency significantly ameliorated lung injury induced by LPS, exogenous supplementation with MRP8/14 could reverse this outcome.

Although endotoxin contaminants were removed by endotoxin removal gel in advance, the biological effects might still be due to LPS contamination. To exclude the effect of unexpected endotoxin contamination, we intravenously injected WT mice with MRP8/14, heat-inactivated (denatured) or recombinant EGFP as a control. The H&E staining results (Figure 1e) showed that the lung injury score (Figure 1f) and W/D ratio (Figure 1g) were elevated by MRP8/14 but not denatured MRP8/14 or EGFP in WT mice. These results suggested that MRP8/14 enhanced lung injury during endotoxemia by promoting the inflammatory response.

### IFNβ mediates MRP8/14-induced *Ip-10* expression in RAW264.7 cells

**Figure 2:**
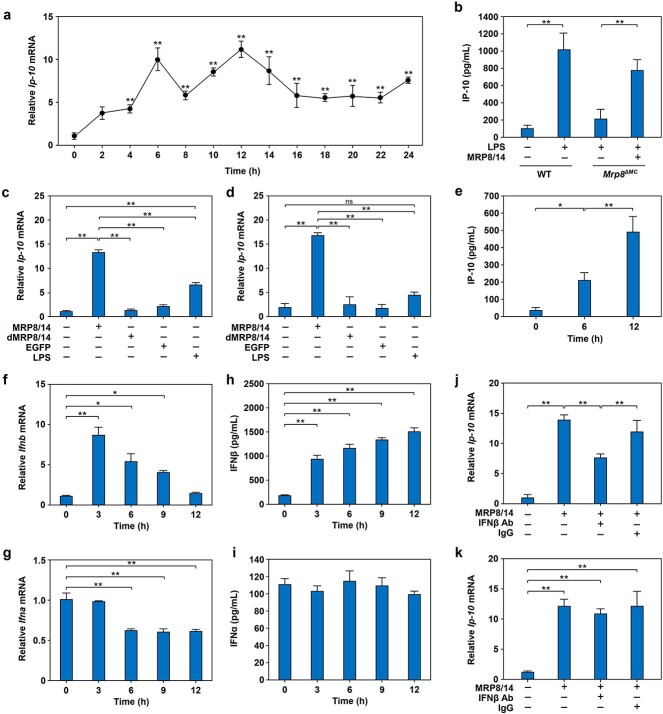
IFNβ mediates MRP8/14-induced *Ip-10* expression in RAW264.7 cells. (**a**) The dynamic profile of *Ip-10* mRNA in RAW264.7 cells treated with MRP8/14 (1.5 μg/ml). *Ip-10* mRNA expression levels were quantified by qPCR. (**b**) IP-10 protein levels in the BALF of WT or *Mrp8*^ΔMC^ mice treated with LPS. WT mice were i.p. injected with LPS (20 mg/kg) or an equal volume of NS. In *Mrp8*^ΔMC^ mice, MRP8/14 (4 mg/kg) or an equal volume of NS was intravenously injected after intraperitoneal LPS administration (20 mg/kg) for 1 h. BALF was collected 12 h after LPS administration, and IP-10 protein levels were quantified by a Luminex multiplex assay. (**c**, **d**) Effect of MRP8/14 on the expression of *Ip-10* mRNA in RAW264.7 cells. RAW264.7 cells were treated with MRP8/14 (1.5 μg/ml), dMRP8/14, EGFP or LPS (100 ng/ml) for 6 h (c) or 12 h (d), and *Ip-10* mRNA expression levels were quantified by qPCR. (**e**) IP-10 protein levels in the supernatants of MRP8/14-treated RAW264.7 cells. RAW264.7 cells were treated with MRP8/14 (1.5 μg/ml) for 0, 6 and 12 h, and IP-10 protein levels in the supernatants were examined by Luminex multiplex assays. (**f**, **g**) MRP8/14 induced *Ifnb* but not *Ifna* mRNA expression in RAW264.7 cells. RAW264.7 cells were treated with MRP8/14 (1.5 μg/ml) for the indicated times (0, 3, 6, 9 and 12 h), followed by quantitation of *Ifnb* (f) and *Ifna* (g) mRNA expression by qPCR. (**h**, **i**) Specific induction of IFNβ protein expression in RAW264.7 cells by MRP8/14. RAW264.7 cells were treated with MRP8/14 (1.5 μg/ml), and IFNβ (h) and IFNα (i) protein levels in the supernatants were quantified by ELISA. (**j**, **k**) IFNβ neutralizing antibody (IFNβ Ab) blocked the induction of *Ip-10* mRNA expression in RAW264.7 cells induced by MRP8/14. RAW264.7 cells were treated with MRP8/14 (1.5 μg/ml) for 12 h (j) or 6 h (k) in the presence or absence of the IFNβ neutralizing Ab (1 μg/ml) or isotype control IgG as a control, followed by *Ip-10* mRNA analysis by qPCR. The data are expressed as mean ± SD and represent three independent experiments (*n* = 3). ^*^p < 0.05, ^*^^*^p< 0.01, ns , not significant. *MRP8/14* myeloid-related protein 8/14, *IP-10* IFNγ inducible protein 10, *IFNβ* interferonβ, *IFNα* interferon α, *qPCR* quantitative real-time polymerase chain reaction, *mRNA* messenger RNA, *BALF* bronchoalveolar lavage fluid, *WT* wild type, *Mrp8*^ΔMC^  *Mrp8* gene-deficient, *NS* normal saline, *LPS* lipopolysaccharide, *dMRP8/14* denatured MRP8/14, *EGFP* enhanced green fluorescent protein, *ELISA* enzyme-linked immunosorbent assay

To examine the dynamic profile of MRP8/14-induced *Ip-10* mRNA expression in RAW264.7 cells, we performed qPCR and found that the expression *of Ip-10* mRNA in RAW264.7 cells was significantly increased at 2 h, peaked twice, at °6 h (early stage) and 12 h (late stage), and was maintained at a high level up to 24 h after treatment with MRP8/14 (Figure 2a). We also found that IP-10 protein levels in the BALF of WT mice was significantly increased after LPS induction for 12 h. In contrast, *Mrp8* gene deficiency significantly blocked the production of IP-10 protein, and this effect could be reversed by exogenous supplementation with MRP8/14 (Figure 2b).

To further exclude the effect of unexpected endotoxin contamination, we treated RAW264.7 cells with MRP8/14, dMRP8/14 or EGFP as a control, and LPS. Unlike MRP8/14 and LPS, dMRP8/14 and EGFP failed to induce *Ip-10* mRNA expression in RAW264.7 cells at 6 (Figure 2c) or 12 h (Figure 2d). Consistently, IP-10 protein expression was found to be induced in RAW264.7 cells after treatment with MRP8/14 for 6 or 12 h (Figure 2e), which suggests that MRP8/14 is involved in IP-10 induction.

Previous studies have demonstrated that IFNs are critical inducers of IP-10 mRNA expression, and we next sought to determine whether IFNs were required for the late-phase expression of IP-10 in MRP8/14-induced macrophages. Notably, *Ifnb* (Figure 2f) but not *Ifna* (Figure 2g) mRNA expression was markedly induced at 3 h by MRP8/14 in macrophages. Correspondingly, the protein expression of IFNβ (Figure 2h) but not IFNα (Figure 2i) in RAW264.7 cells was specifically induced by MRP8/14. Interestingly, blockade with a neutralizing Ab against IFNβ (Neutralizing activity assay of IFNβ antibody see supplementary Figure S3) significantly reduced *Ip-10* mRNA expression in MRP8/14-treated RAW264.7 cells for 12 h (Figure 2j) but not 6 h (Figure 2k), indicating that IFNβ mediates late-phase induction of IP-10 expression in MRP8/14-treated RAW264.7 cells.

### The JAK–STAT signaling pathway regulates *Ip-10* gene expression in MRP8/14-treated RAW264.7 cells

**Figure 3:**
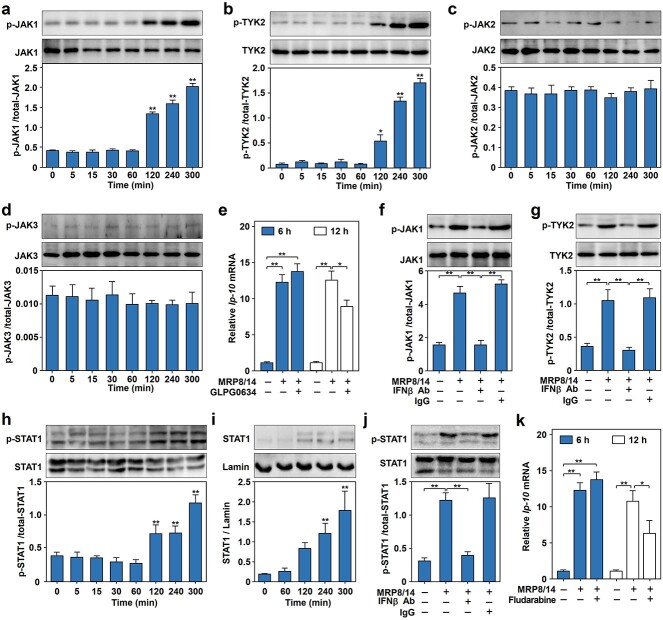
The JAK–STAT signaling pathway is involved in the regulation of *Ip-10* gene expression in MRP8/14-treated RAW264.7 cells. (**a**–**d**) JAK1 and TYK2 but not JAK2 or JAK3 were phosphorylated in MRP8/14-treated RAW264.7 cells. RAW264.7 cells were treated with MRP8/14 (1.5 μg/ml) for the indicated times (0, 5, 15, 30, 60, 120, 240 and 300 min), followed by total cellular protein extraction and immunoblot analysis of phosphorylated/total JAK1 (a), TYK2 (b), JAK2 (c) and JAK3 (d). The protein levels were quantified as the relative intensity of the protein bands on the blots. (**e**) The JAK1 inhibitor GLPG0634 blocked *Ip-10* mRNA expression in RAW264.7 cells induced by MRP8/14. RAW264.7 cells were pretreated with or without GLPG0634 (500 nM) for 2 h and then stimulated with MRP8/14 (1.5 μg/ml) for 6 or 12 h. The mRNA expression levels of *Ip-10* were quantified by qPCR. (**f**, **g**) IFNβ neutralizing Ab blocked the phosphorylation of JAK1 and TYK2 in RAW264.7 cells induced by MRP8/14. After pretreatment with the IFNβ neutralizing Ab (1 μg/ml) or isotype control IgG for 2 h, RAW264.7 cells were treated with MRP8/14 (1.5 μg/ml) for 5 h. Phosphorylated and total JAK1 (f) and TYK2 (g) were examined by immunoblotting with specific antibodies. The protein levels were quantified as the relative intensity of the protein bands on the blots. (**h**) Dynamic profile of STAT1 phosphorylation in MRP8/14-treated RAW264.7 cells. RAW264.7 cells were stimulated with MRP8/14 (1.5 μg/ml) for the indicated times (0, 5, 15, 30, 60, 120, 240 and 300 min), followed by total cellular protein extraction and immunoblot analysis of phosphorylated and total STAT1. The protein levels were quantified as described above. (**i**) Time-dependent STAT1 nuclear translocation in MRP8/14-treated RAW264.7 cells. RAW264.7 cells were stimulated with MRP8/14 (1.5 μg/ml) for the indicated times (0, 60, 120, 240, 300 min), followed by total nuclear protein extraction and immunoblot analysis of total STAT1 and lamin as a control. The protein levels were quantified as described above. (**j**) The IFNβ neutralizing Ab blocked the phosphorylation of STAT1 in RAW264.7 cells treated with MRP8/14. After pretreatment with the IFNβ neutralizing Ab (1 μg/ml) or isotype control IgG for 2 h, RAW264.7 cells were treated with MRP8/14 (1.5 μg/ml) for 5 h. Phosphorylated and total STAT1 were examined by immunoblotting with specific Abs. The protein levels were quantified as the relative intensity of the protein bands on the blots. (**k**) The STAT1 inhibitor fludarabine blocked *Ip-10* mRNA expression in RAW264.7 cells induced by MRP8/14. RAW264.7 cells were pretreated with or without fludarabine (10 μM) for 2 h and then stimulated with MRP8/14 (1.5 μg/ml) for 6 or 12 h. The mRNA expression levels of *Ip-10* were quantified by qPCR. The data are expressed as the mean ± SD and represent three independent experiments (*n* = 3). ^*^p < 0.05, ^*^^*^p < 0.01. *MRP8/14* myeloid-related protein 8/14, *IP-10* IFNγ inducible protein 10, *JAK1* Janus kinase 1, *p-JAK1* phosphorylated Janus kinase 1, *JAK2* Janus kinase 2, *p-JAK2* phosphorylated Janus kinase 2, *JAK3* Janus kinase 3, *p-JAK3* phosphorylated Janus kinase 3, *TYK2* tyrosine kinase 2, *p-TYK2* phosphorylated tyrosine kinase 2, *GLPG0634* JAK1 inhibitor, *IFNβ Ab* interferonβ neutralizing antibody, *STAT1* signal transducer and activator of transcription 1, *p-STAT1* phosphorylated signal transducer and activator of transcription 1, *qPCR* quantitative real-time polymerase chain reaction, *mRNA* messenger RNA

To clarify the involvement of the JAK–STAT pathway in *Ip-10* gene induction, we first examined the phosphorylation of JAK1, JAK2, JAK3 and TYK2 and found that JAK1 (Figure 3a) and TYK2 (Figure 3b) but not JAK2 (Figure 3c) or JAK3 (Figure 3d) were highly phosphorylated in RAW264.7 cells after MRP8/14 treatment for 120–300 min. Next, we used GLPG0634 to inhibit the activity of JAK1 and found that *Ip-10* mRNA expression in RAW264.7 cells was significantly blocked at 12 h but not 6 h after MRP8/14 administration (Figure 3e), indicating that the activation of JAK1/TYK2 was involved in the late-phase induction of *Ip-10* by MRP8/14. In addition, inhibiting IFNβ using an IFNβ neutralizing Ab significantly inhibited the MRP8/14-induced phosphorylation of both JAK1 (Figure 3f) and TYK2 (Figure 3g), suggesting that MRP8/14 triggers the phosphorylation of JAK1 and TYK2 via IFNβ released into the extracellular space.

To examine the role of STAT1 in *Ip-10* expression in MRP8/14-induced macrophages, we performed western blotting with phosphorylation-specific Abs and found that the phosphorylation of STAT1 was significantly elevated in RAW264.7 cells treated with MRP8/14 for 120–300 min (Figure 3h). Consistent with this result, STAT1 significantly translocated into the nucleus in macrophages following treatment with MRP8/14 (Figure 3i). In addition, we found that blocking IFNβ with an IFNβ neutralizing Ab significantly reduced the MRP8/14-induced phosphorylation of STAT1 (Figure 3j), whereas the STAT1 inhibitor fludarabine suppressed *Ip-10* mRNA expression in RAW264.7 cells after MRP8/14 treatment for 12 h (Figure 3k). These results indicated that IFNβ mediated late-phase *Ip-10* induction by MRP8/14 in RAW264.7 cells via the JAK–STAT signaling pathway.

### IRF7 drives *Ip-10* gene transcription in macrophages induced by MRP8/14

**Figure 4:**
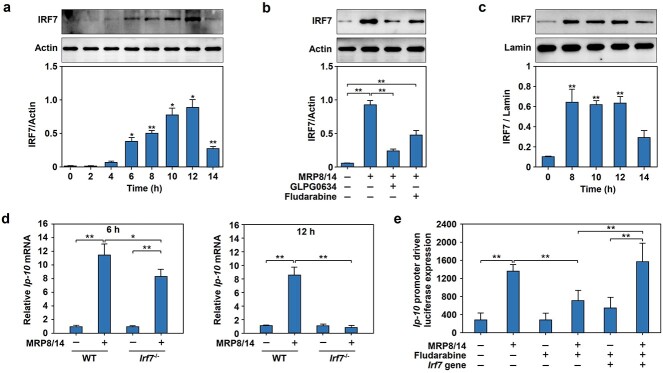
IRF7 functions downstream of the JAK–STAT pathway to mediate MRP8/14-induced *Ip-10* gene expression in macrophages. (**a**) Profile of IRF7 protein expression in MRP8/14-treated RAW264.7 cells. RAW264.7 cells were treated with MRP8/14 (1.5 μg/ml) for the indicated times (0, 2, 4, 6, 8, 10, 12 and 14 h), followed by total cellular protein extraction and immunoblot analysis of IRF7. The protein levels were quantified as described above. (**b**) Inhibition of the JAK–STAT pathway reduced IRF7 production in RAW264.7 cells induced by MRP8/14. After pretreatment with GLPG0634 (500 nM) or fludarabine (10 μM) for 2 h, RAW264.7 cells were treated with MRP8/14 (1.5 μg/ml) for 12 h. IRF7 protein expression was quantified as described above. (**c**) Nuclear translocation of IRF7 in MRP8/14-treated RAW264.7 cells. RAW264.7 cells were treated with MRP8/14 (1.5 μg/ml) for the indicated times (0, 8, 10, 12and 14 h), followed by nuclear protein extraction and immunoblot analysis of IRF7. Lamin was used as a control and the protein levels were quantified as described above. (**d**) *Irf7* gene deficiency reduced late-stage *Ip-10* mRNA expression in BMDMs induced by MRP8/14. BMDMs were isolated from WT or *Irf7*^−/−^ mice and treated with or without MRP8/14 (1.5 μg/ml) for 6 or 12 h. Total RNA was extracted from BMDMs by the TRIzol method and *Ip-10* mRNA expression was quantified by qPCR. (**e**) *Irf7* gene overexpression rescued the transcriptional activity of *Ip-10* that was downregulated by STAT1 inhibitor in MRP8/14-treated RAW264.7 cells. After transfection with the *Ip-10* reporter (PGL3-*Ip10p*) with or without the *irf7*-expressing plasmid (pcDNA3-*Irf*7), RAW264.7 cells were cultured for 24 h. Then, the cells were incubated with fludarabine (10 μM) for 2 h and treated with MRP8/14 (1.5 μg/ml) for 12 h. Cell lysates were extracted for the dual-luciferase assay, and relative luciferase activities were calculated by determining the ratio of firefly and Renilla luciferase activities. The data are expressed as mean ± SD and represent three independent experiments (*n* = 3). ^*^p < 0.05, ^*^^*^  *p <* 0.01. *IRF7* interferon regulatory factor-7, *MRP8/14* myeloid-related protein 8/14, *IP-10* IFNγ inducible protein 10, *JAK* Janus kinase, *STAT* signal transducer and activator of transcription, *GLPG0634* JAK1 inhibitor, *WT* wild-type, *Irf7*^−/−^  *Irf7* gene deficiency, *BMDMs* bone marrow-derived macrophages, *qPCR* quantitative real-time polymerase chain reaction, *mRNA* messenger RNA

IRF7 was reported to be a downstream target of IFNβ; thus, we performed western blotting to examine whether IRF7 was involved in MRP8/14-induced *Ip-10* gene expression in macrophages. The results showed that IRF7 protein expression significantly increased at 6 h and peaked at 12 h in RAW264.7 cells after treatment with MRP8/14 (Figure 4a). To address whether the induction of IRF7 in MRP8/14-induced macrophages occurs through the JAK–STAT pathway, we used JAK1 and STAT1 inhibitors to block this signaling pathway and found that inhibiting JAK1 or STAT1 significantly suppressed IRF7 production in RAW264.7 cells (Figure 4b). Nuclear translocation is necessary for a transcription factor to activate target gene transcription; we examined whether IRF7 was localized in the nucleus in macrophages and found that IRF7 translocated into the nucleus in MRP8/14-treated RAW264.7 cells from 8 to 12 h (Figure 4c).

To determine whether IRF7 was necessary in the late-phase induction of *Ip-10* expression, BMDMs from WT and *Irf7*-deficient (*Irf7^−/−^*) mice were prepared and treated with MRP8/14. The results showed that the mRNA expression of *Ip-10* was markedly increased in BMDMs derived from both WT and *Irf7^−/−^* mice treated with MRP8/14 for 6 h, accompanied by slight suppression of *Ip-10* expression in BMDMs derived from *Irf7^−/−^* mice. In contrast to WT mice, *Irf7^−/−^* mice failed to show notable accumulation of *Ip-10* mRNA in BMDMs after treatment with MRP8/14 for 12 h, suggesting that IRF7 is required for *Ip-10* transcription in the late stage rather than in the early stage (Figure 4d). We further used a luciferase reporter system to examine the role of STAT1 in IRF7-mediated modulation of the transactivation of the *Ip-10* promoter. Consistent with previous results, the STAT1 inhibitor fludarabine markedly suppressed MRP8/14-induced transactivation of *Ip-10*. Intriguingly, overexpression of the *Irf7* gene fully restored the transactivation activity of the *Ip-10* promoter in MRP8/14-treated RAW264.7 cells (Figure 4e).

**Figure 5:**
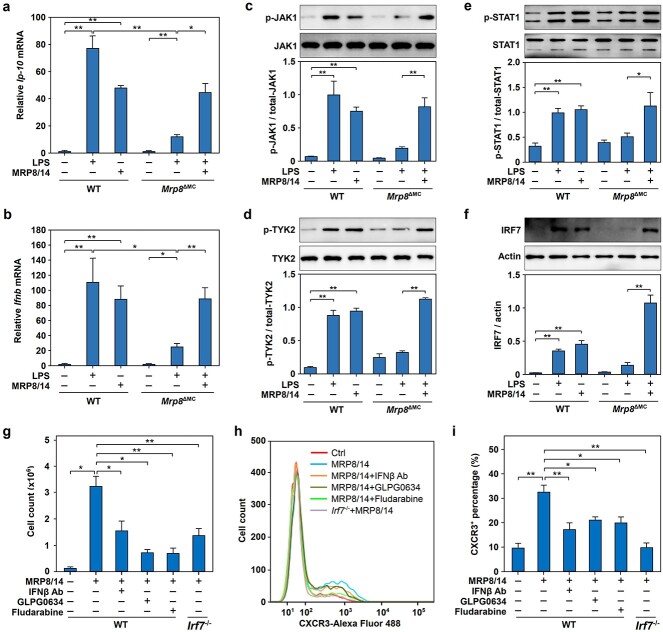
Chemotaxis of CXCR3^+^ lymphocytes was induced by activation of the IFNβ-JAK1/TYK2-STAT1-IRF7 pathway *in vivo.* (**a**, **b**) *Mrp8* gene deficiency reduces the expression of *Ip-10* and *Ifnb* mRNAs in endotoxic mice. WT mice were treated with LPS (20 mg/kg, i.p.), MRP8/14 (4 mg/kg, i.v.) or an equal volume of NS. *Mrp8*^ΔMC^ mice were treated with LPS, LPS plus MRP8/14, or an equal volume of NS. After treatment with LPS or MRP8/14 for 12 h, AMs were isolated, and *Ip-10* (a) and *Ifnb* (b) mRNA expression was quantified by qPCR. (**c**–**e**) Effect of *Mrp8* gene deficiency on the phosphorylation of JAK1, TYK2 and STAT1 in AMs induced by LPS. After treatment with LPS or MRP8/14 for 12 h, AMs were isolated from mice, phosphorylated and total JAK1 (c), TYK2 (d) and STAT1 (e) levels were quantified by immunoblotting. The protein levels were quantified by determining the relative intensity of the protein bands on the blots. (**f**) Effect of *Mrp8* gene deficiency on IRF7 expression in AMs treated with LPS. AMs were isolated from mice treated with LPS for 12 h and immunoblotting was performed to quantify IRF7 protein expression. (**g**–**i**) The IFNβ-JAK1/TYK2-STAT1-IRF7 signaling pathway was involved in the transmigration of CXCR3^+^ lymphocytes induced by MRP8/14 *in vivo*. After pretreatment with or without the IFNβ neutralizing Ab (1 μg/ml), GLPG0634 (500 nM) or fludarabine (10 μM) for 2 h, BMDMs from WT or *Irf7*^−/−^ mice were treated with MRP8/14 (1.5 μg/ml) for 36 h, and cell culture supernatants were collected for the air pouch assay. After preparation of the air pouches *in vivo* for 5 days, the mice were i.p. administered LPS for 8 h. Then, the cell culture supernatants were injected into the air pouches and the lavage fluids were collected from the air pouches for further analysis. Wright–Giemsa staining was performed and the total number of migrated lymphocytes was counted (g). Flow cytometry was performed to analyse the proportion of CXCR3^+^ lymphocytes among all the migrated cells (h, i). The data are expressed as mean ± SD and represent three independent experiments (*n* = 3). ^*^p < 0.05, ^*^^*^p < 0.01. *CXCR3* chemokine (C-X-C motif) receptor 3*, MRP8/14* myeloid-related protein 8/14, *IP-10* IFNγ inducible protein 10, *WT* wild-type, *Irf7*^−/−^  *Irf7* gene deficiency, *Mrp8*^ΔMC^  *Mrp8* gene-deficient, *LPS* lipopolysaccharide, *NS* normal saline, *IFNβ* interferonβ, *JAK1* Janus kinase 1, *TYK2* tyrosine kinase 2, *STAT1* signal transducer and activator of transcription 1, *IRF7* interferon regulatory factor-7, *GLPG0634* JAK1 inhibitor, *IFNβ Ab* interferonβ neutralizing antibody, *AMs* alveolar macrophages*, BMDMs* bone marrow-derived macrophages, *qPCR* quantitative real-time polymerase chain reaction, *mRNA* messenger RNA

These results suggested that IRF7 functions as a downstream molecule of the JAK–STAT pathway to transactivate *Ip-10* gene expression in MRP8/14-induced macrophages.

**Figure 6:**
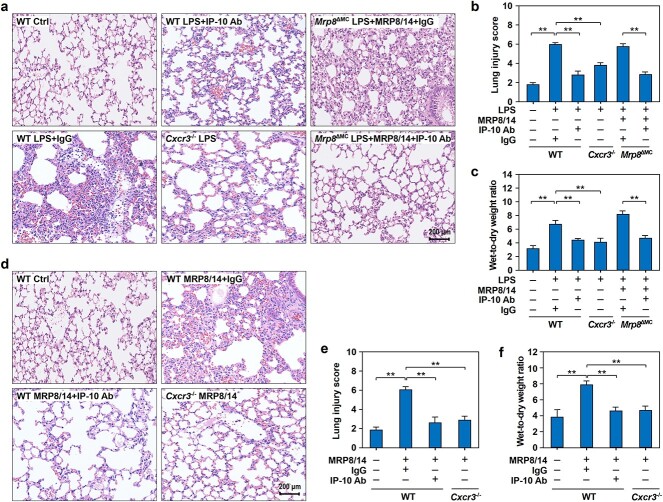
IP-10 blockade and *Cxcr3* gene deficiency alleviated lung injury in endotoxic mice. (**a**–**c**) Effect of IP-10 blockade and *Cxcr3* gene deficiency on lung injury in endotoxic mice. WT mice were injected i.p. with NS or LPS (20 mg/kg) in the presence of IP-10 neutralizing antibody (IP-10 Ab) (2 mg/kg body weight) or equal amounts of isotype control IgG. *Cxcr3^−/−^* mice were injected i.p. with LPS (20 mg/kg). *Mrp8*^ΔMC^ mice were challenged with LPS plus MRP8/14 in the presence of IP-10 Ab or equal amounts of IgG. H&E staining was performed to determine the histopathological changes in lung tissues from mice treated with LPS for 12 h and representative histopathological images are shown with a scale bar of 200 μm (a). Lung injury scores were determined by semiquantitative lung injury analysis (b). The W/D ratio of lung tissues from mice in the different groups was calculated (c). (**d**–**f**) Effect of IP-10 blockade and *Cxcr3* gene deficiency on MRP8/14-induced lung injury in mice. WT mice were i.v. injected with MRP8/14 (4 mg/kg) in the presence of IP-10 Ab or equal amounts of IgG. *Cxcr3*^−/−^ mice were treated with MRP8/14 alone. After MRP8/14 treatment for 12 h, lung tissues were collected from WT or *Cxcr3*^−/−^ mice. H&E staining was performed to determine histopathological changes in lung tissues and representative histopathological images are shown with a scale bar of 200 μm (d). Lung injury scores were determined as described above (e). W/D ratio was calculated as described above (f). The data are expressed as mean ± SD and represent three independent experiments (*n* = 3). ^*^p < 0.05, ^*^^*^p < 0.01. *MRP8/14* myeloid-related protein 8/14, *IP-10* IFNγ inducible protein 10, *WT* wild-type, *Cxcr3*^−/−^  *Cxcr3* gene deficiency, *Mrp8*^ΔMC^  *Mrp8* gene-deficient, *LPS* lipopolysaccharide, *NS* normal saline, *H&E* hematoxylin and eosin, *W/D* wet-to-dry

### IFNβ-JAK1/TYK2-STAT1-IRF7 pathway is required for CXCR3^+^ T lymphocyte migration in endotoxemia

To determine the contribution of MRP8/14 to LPS-induced *Ip-10* and *Ifnb* mRNA expression *in vivo,* AMs were isolated from WT or *Mrp8*^ΔMC^ mice, and the qPCR results showed that LPS and MRP8/14 dramatically induced the mRNA expression of *Ip-10* (Figure 5a) and *Ifnb* (Figure 5b) in AMs from WT mice. Interestingly, LPS failed to induce the expression of either mRNA in AMs from *Mrp8* gene-deficient mice, and this effect was restored by the administration of exogenous MRP8/14 (Figure 5a, b).

To further determine the contribution of MRP8/14 to the activation of the JAK1/TYK2-STAT1-IRF7 pathway, we performed western blotting with phosphorylation-specific Abs and found that the phosphorylation of JAK1 (Figure 5c), TYK2 (Figure 5d) and STAT1 (Figure 5e) were enhanced by MRP8/14, similar to the effect of LPS; *Mrp8* gene deficiency significantly reduced the phosphorylation of JAK1, TYK2 and STAT1 in AMs induced by LPS, and the administration of MRP8/14 could restore the phosphorylation of JAK1, TYK2 and STAT1 in *Mrp8*^ΔMC^ mice (Figure 5c–e). Similarly, we also found that *Mrp8* gene deficiency significantly reduced the expression of IRF7 induced by LPS and that MRP8/14 could restore IRF7 expression in AMs from *Mrp8*^ΔMC^ mice (Figure 5f). These results indicated that MRP8/14 induced *Ip-10* mRNA expression in the late stage of endotoxemia by activating the IFNβ-JAK1/TYK2-STAT1-IRF7 signaling pathway.

To evaluate the role of the IFNβ-JAK1/TYK2-STAT1-IRF7 pathway in lymphocyte migration during endotoxemia, we established an *in vivo* air pouch model and found that supernatants of BMDMs treated with MRP8/14 significantly enhanced the chemotaxis of white blood cells, whereas the supernatants of BMDMs treated with the IFNβ neutralizing Ab, GLPG0634 or fludarabine or from *Irf7*^−/−^ mice failed to increase the chemotaxis of white blood cells into the air pouches (Figure 5g). We further performed flow cytometry to examine the CXCR3^+^ T lymphocyte proportion among total migrated cells and found that the proportion of CXCR3^+^ T lymphocytes was significantly increased in the air pouches that were injected with the supernatants from BMDMs treated with MRP8/14. This effect was largely restrained by blocking the signaling pathway with GLPG0634 or fludarabine, neutralizing IFNβ with a specific Ab or *Irf7* gene deficiency (Figure 5h and i).

Taken together, these results indicate that MRP8/14-induced migration of CXCR3^+^ T lymphocytes in endotoxemic mice is dependent, at least in part, on activation of the IFNβ-JAK1/TYK2-STAT1-IRF7 pathway.

### IP-10–CXCR3 interactions promote lung endotoxemic injury

To further characterize the role of IP-10–CXCR3 in lung injury in the context of endotoxemia, we performed histopathological evaluation with a neutralizing Ab against IP-10 and *Cxcr3*-deficient mice. The results showed that IP-10 neutralization and *Cxcr3* gene deficiency significantly reduced LPS-induced lung injury (Figure 6a–c).

The experiments with *Mrp8^ΔMC^* mice showed that LPS-induced lung injury was largely dependent on the expression of *Mrp8 in vivo* (Figure 1b–d); thus, we examined whether IP-10 neutralization also had a therapeutic effect on MRP8/14-induced lung injury and found that targeting IP-10 with a neutralizing Ab significantly reduced lung injury in *Mrp8^ΔMC^* mice treated with LPS plus MRP8/14 (Figure 6a–c).

To further clarify the importance of the interaction between IP-10 and CXCR3 in lung injury induced by MRP8/14, we examined the biological consequences of IP-10 neutralization and *Cxcr3* deficiency in mice treated with MRP8/14 and found that MRP8/14-induced lung injury was significantly reduced in WT mice treated with an IP-10 neutralizing Ab, as well as in *Cxcr3^−/−^* mice (Figure 6d–f).

## Discussion

**Figure 7:**
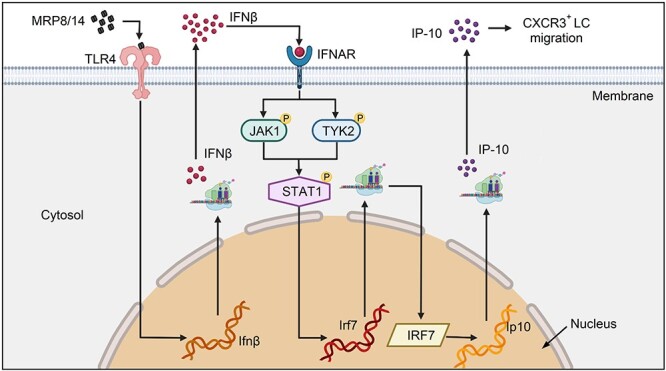
Signaling model for late-phase IP-10 induction by MRP8/14 in endotoxic mice. At the beginning of endotoxemia, MRP8/14 is released from activated neutrophils into the blood. Extracellular MRP8/14 binds to TLR4 on the surface of macrophages to induce IFNβ production and release. Then, IFNβ activates the JAK1/TYK2-STAT1 signaling pathway by binding with IFNAR, resulting in *irf7* gene transcription and IRF7 protein synthesis. As a transcription factor, IRF7 promotes *Ip-10* gene transcription, resulting in sustained production of IP-10 in the late phase of endotoxemia. Finally, the accumulated IP-10 attracts a large number of CXCR3^+^ lymphocytes to the site of infection, leading to an over-reactive inflammatory response and lung injury. *MRP8/14* myeloid-related protein 8/14, *IP-10* IFNγ inducible protein 10, *IFNβ* interferon β, *JAK1* Janus kinase 1, *TYK2* tyrosine kinase 2, *STAT1* signal transducer and activator of transcription 1, *IRF7* interferon regulatory factor-7, *CXCR3* chemokine (C-X-C motif) receptor 3, *IFNAR* type I interferon receptor

Extracellular MRP8/14, which is a heterodimeric complex released by phagocytes during infection, acts as an endogenous alarmin that amplifies the TLR4 signaling-dependent inflammatory response by inducing proinflammatory cytokines, such as TNF-α, IL-1β, IL-12 and IL-18 [9,12]. However, the pathological functions of MRP8/14 in endotoxemia have not yet been fully elucidated. In this study, we demonstrated that MRP8/14 induces sustained IP-10 expression via the IFNβ-JAK1/TYK2-STAT1-IRF7 pathway, which is involved in lung injury during endotoxemia (Figure 7).

Previous studies have shown that MRP8, but not MRP14, is the active component of the MRP8/14 dimer [9]; however, in most studies, *Mrp14*^−/−^ mice have been used to examine the function of MRP8/14 [9,16,34]. Thus, it is important to fully examine the effect of *Mrp8* deficiency on the pathological process of endotoxemia. Since conventional knockout of the *Mrp8* gene in mice is embryonically lethal [35], we used myeloid cell-specific *Mrp8* gene-deficient mice to conduct this study.

A previous study demonstrated that the MRP8–MRP14 complex acts as an endogenous activator of TLR4 to amplify phagocyte activation, promoting lethal, endotoxin-induced shock [9,36]. Consistent with this result, our findings showed that myeloid cell-specific deletion of the *Mrp8* gene attenuated the inflammatory response and lung injury in endotoxemic mice, and this effect could be reversed by exogenous recombinant MRP8/14, indicating that MRP8/14 is involved in the inflammatory response and contributes to lung injury in endotoxemia.

It is important to address how extracellular MRP8/14 promotes the LPS-induced inflammatory response and lung injury. One explanation is that MRP8/14, which serves as a damage-associated molecular pattern, enhances the production of proinflammatory cytokines, such as TNF-α, to mediate lethal endotoxemia [9,37]. However, clinical trials have shown that blocking TNF-α is harmful rather than beneficial in patients with sepsis [38,39], suggesting that other proinflammatory factors induced by MRP8/14 are critical for organ injury in endotoxemia.

As a chemokine associated with activated CXCR3^+^ T lymphocytes, IP-10 is highly induced in macrophages in Th1-type inflammatory diseases [40,41]. Our study showed that *Mrp8* gene deficiency significantly blocked LPS-induced IP-10 expression and that the administration of exogenous MRP8/14 restored IP-10 induction in BALF; blocking IP-10 restrained lung injury in endotoxemic mice, indicating that MRP8/14 was a key inducer of IP-10, which is crucial for the inflammatory response and lung injury during endotoxemia.

Previous studies have shown that IP-10 is increased in both serum [22] and BALF [42] during endotoxic shock; however, the role of IP-10 in inflammation is controversial. Some researchers have suggested that IP-10 is beneficial in infectious diseases [43–45]. For example, in *Toxoplasma gondii* infection, IP-10 is involved in the migration of activated T cells, which controls tissue parasite burden and increases the survival of infected mice [43]. On the other hand, IP-10 has been suggested to have a close association with the inflammatory response and histopathological injury during infection [46–48]. For example, during chronic infection with hepatitis C virus, IP-10 expression in hepatocytes attracts inflammatory T cells to the liver lobule, thus inducing liver injury [46,48].

Because inflammation is a double-edged sword, the biological consequence of IP-10 might be time- and dose-dependent. Although early induction of IP-10 is helpful in the infiltration of CXCR3^+^ T lymphocytes and sensing pathogen infection, sustained overexpression of IP-10 in the late stage of endotoxemia resulted in severe lung injury, indicating the importance of IP-10 as an activator that amplifies the proinflammatory response in endotoxemia. The recruitment of inflamed CXCR3^+^ T cells provides a reasonable explanation for IP-10-induced histopathological injury in endotoxemic mice. As a functional ligand for the CXCR3 receptor, IP-10 is highly expressed in activated T lymphocytes, including proinflammatory and regulatory T cells [40,49–52]. In severe respiratory viral infection, IP-10 was reported to attract inflamed CXCR3^+^ neutrophils, resulting in fulminant pulmonary inflammation [47]. In hepatitis C virus infection, IP-10 was reported to recruit inflammatory CXCR3^+^ T lymphocytes to enhance the inflammatory response [46,48]. However, our results showed that both IP-10 neutralization and deletion of the *Cxcr3* gene could reduce lung injury in endotoxemic mice, indicating that massive IP-10 induction by MRP8/14 increases the infiltration of CXCR3^+^ T lymphocytes, subsequently leading to severe lung injury.

In the present study, we found that IFNβ was involved in sustained *Ip-10* production in MRP8/14-induced macrophages; however, the underlying mechanism of IP-10 induction in the context of endotoxemia is unclear. As a type I IFN member, IFNβ is induced during the early response to LPS and is responsible for the lethality of LPS-induced endotoxic shock [53,54]. IFNβ has also been reported to induce proinflammatory cytokines, including macrophage inflammatory protein-1β (MIP-1β), regulated on activation, normal T cell expressed and secreted (RANTES) and IP-10 [55,56].

Our findings clearly demonstrated that activation of the JAK1/TYK2-STAT1 pathway by IFNβ was involved in IP-10 production in the late-phase response to MRP8/14. As a transcription factor, STAT1 directly drives the transcription of interferon-stimulated genes, including IP-10, by binding to *cis* elements in their promoter sequence [57]. In this study, STAT1 was activated and translocated into the nucleus in RAW264.7 cells after exposure to MRP8/14 for 2–5 h, while the effects of suppressing *Ip-10* gene expression with GLPG0634 mainly manifested after treatment with MRP8/14 for 12 h, indicating that the accumulation of *Ip-10* mRNA in the late stage of MRP8/14 treatment is not directly mediated by the JAK–STAT pathway but is mediated by another downstream factor. Previous studies demonstrated that IRF7 is strongly induced by type I IFN-mediated signaling in a manner that is dependent on TYK2-mediated phosphorylation of Tyr-701 of STAT1, suggesting that *Irf7* is a downstream target of STAT1 [58]. We found that the transcriptional activity of the *Ip-10* promoter was significantly suppressed by the STAT1 inhibitor fludarabine and that *Irf7* could ameliorate the inhibition of *Ip-10* promoter transactivation, suggesting that *Irf7* is a downstream regulator of the JAK–STAT pathway that induces *Ip-10* transcription in the late stage of MRP8/14 treatment in macrophages. However, how IRF7 drives *Ip-10* transcription is unclear. IRFs have been reported to serve as transcription factors that mediate interferon-stimulated gene transcription by binding to interferon-stimulated response element (ISRE) consensus sequences [59]. To date, three ISRE sites have been identified in the promoter sequence of *Ip-10* [60,61]. IRF1 can bind to an ISRE sequence in the promoter to induce *Ip-10* gene expression in 2fTGH cells treated with IFNγ/TNF-α [60]. Our previous study demonstrated that IRF3 binds to an ISRE site to induce *Ip-10* gene transcription in macrophages treated with MRP8/14 [17]. These results suggest that the binding of IRFs to the ISRE sequence in the promoter is critical for *Ip-10* gene transcription in different contexts. Thus, it is reasonable to propose that IRF7 induces *Ip-10* gene transcription by binding to ISRE sites.

## Conclusions

MRP8/14 induces sustained IP-10 production by activating the IFNβ-JAK1/TYK2-STAT1-IRF7 pathway, which subsequently attracts numerous CXCR3^+^ T cells and results in an exaggerated inflammatory response and lung injury in the context of endotoxemia. Our findings shed new light on the critical role of the IFNβ-JAK1/TYK2-STAT1-IRF7 pathway in MRP8/14-induced IP-10 expression in the progression of endotoxemia, which may provide new therapeutic strategies for sepsis.

**Abbreviations**
Ab: Antibody; Actb: β actin; AM: Alveolar macrophage; ANOVA: analysis of variance; BALF: Bronchoalveolar lavage fluid; BMDM: bone marrow-derived macrophage; BSA: bovine serum albumin; dMRP: Denatured myeloid-related protein; EGFP: Enhanced green fluorescent protein; ELISA: Enzyme-linked immunosorbent assay; H&E: Hematoxylin and eosin; IFNAR: typeIinterferon receptor; IFN-γ: Interferon γ; IL-1β: Interleukin-1β; i.p.: Intraperitoneally; IP-10: Interferon γ-inducible protein 10; i.v.: Intravenously; ISRE: Interferon-stimulated response element; LPS: Lipopolysaccharide; MCP-1: Monocyte chemoattractant protein-; MIP-1b: macrophage inflammatory protein-1β; MRP: Myeloid-related protein; NS: Normal saline; qPCR: Quantitative real-time polymerase chain reaction; mIFNβ: mouse interferonβ; RANTES: regulated on activation, normal T cell expressed and secreted; TNF-α: Tumor necrosis factor α; TLR4: Toll-like receptor 4; W/D: Wet-to-dry weight; WT: Wild-type.

## Supplementary Material

Supplementary_File_tkad006

## Data Availability

The data used to support the findings of this study are available from the corresponding author upon request.
