# Supplementary material for: Sustained induction of IP-10 by MRP8/14 via the IFNβ–IRF7 axis in macrophages exaggerates lung injury in endotoxemic mice
Source: Burns Trauma. 2023 Sep 11;11:tkad006. doi: 10.1093/burnst/tkad006 (PMC10494486; doi:10.1093/burnst/tkad006)
Supplement: Supplementary_File_tkad006 [file supplementary_file_tkad006.pdf]

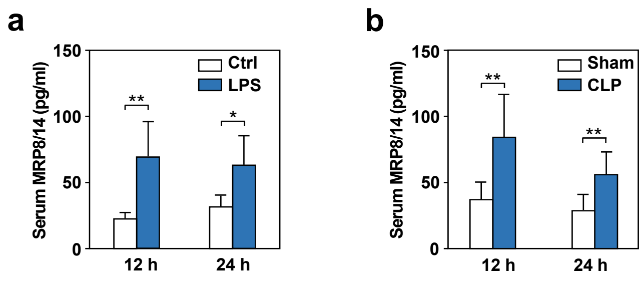

Figure S1. Elevation of MRP8/14 in the sera of mice subjected to LPS injection or cecal ligation and puncture (CLP) modeling.

(a) Quantitation of MRP8/14 in the sera of mice subjected to LPS injection. WT mice were intraperitoneally injected with LPS (20 mg/kg) or normal saline (NS) as control. Serum was collected 12 h or 24 h after administration of LPS or NS, followed by ELISA quantitation of MRP8/14. **(b)** Quantitation of MRP8/14 in the sera of CLP mice. Serum was collected from WT mice 12 h or 24 h after CLP modeling or sham operation for the measurement of MRP8/14 by ELISA. The data are expressed as the mean ± SD and represent six independent experiments (n=6). * P<0.05, ** P<0.01. *MRP8/14* myeloid-related protein 8/14, *LPS* lipopolysaccharide, *NS* normal saline, *WT* wild-type, *ELISA* enzyme-linked Immunosorbent assay.

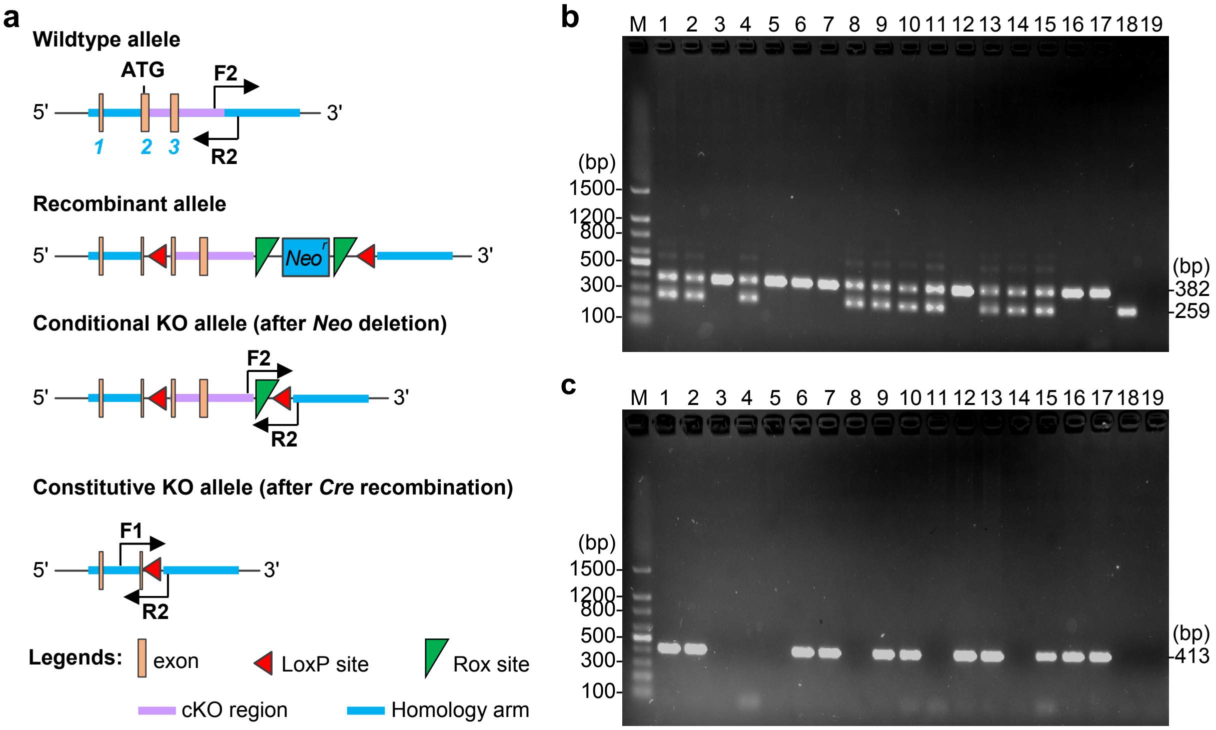

Figure S2. Generation and identification of mice with *Mrp8* deletion in myeloid lineage cells.

(a) Schematic diagram of the establishment of *Mrp8* conditional knockout. Mice with *Mrp8* deletion in myeloid lineage cells were generated by crossing *Mrp8*$^{loxP/loxP}$ mice with *Lyz2-Cre* knockin mice. **(b)** Identification of mice with the *loxP/loxP* genotype (PCR product length, 382 bp). The lengths of the PCR products from the WT and heterozygous mice were 259 bp and 413 bp, respectively. **(c)** Identification of mice with the *Lyz2-Cre*$^{+}$ genotype (PCR product length, 413 bp). *Mrp8* myeloid-related protein 8, *KO* knock out, *WT* wild-type, *PCR* polymerase chain reaction, *Cre* causes recombination, *LoxP* locus of X-overP1.

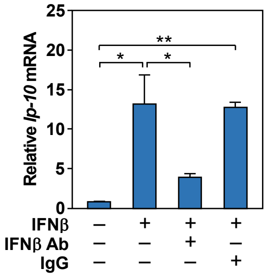

Figure S3. The IFNβ Ab blocked the induction of *Ip-10* mRNA expression in RAW264.7 cells induced by IFNβ.

Raw264.7 cells were pretreated with 1 μg/ml IFNβ neutralized Ab (IFNβ Ab) or isotype control for 2 h and then stimulated with mouse IFNβ (10 ng/ml) for 12 h. *Ip-10* mRNA were quantified by qPCR. The data are expressed as the mean ± SD and represent three independent experiment (n=3). * P<0.05, ** P<0.01. *IFNβ* interferonβ, *Ip-10* IFNγ inducible protein 10, *qPCR* quantitative real-time polymerase chain reaction, *mRNA* messenger RNA.

Supplementary Table 1. Primer sequences for qPCR

Gene name	Primer	Sequence (5'-3')
Ip-10	Forward	GCCGTCATTTTCTGCCTCAT
	Reverse	GCTTCCCTATGGCCCTCATT
Cxcr3	Forward	GGTTAGTGAACGTCAAGTGCT
	Reverse	CCCCATAATCGTAGGGAGAGGT
Ifna	Forward	ATGAGCACTGAAAGCATGATC
	Reverse	TCACAGGGCAATGATCCCAAAGTAGACCTGCCC
Ifnb	Forward	ATGACACCACCTGAACGTCTCTTC
	Reverse	CTACAGAGCGAAGGCTCCAAAGAAGACAGTACT
Actb	Forward	AGCGAGCATCCCCCAAAGTT
	Reverse	GGGCACGAAGGCTCATCATT

Ip-10 IFNγ inducible protein 10, *Cxcr3* chemokine (C-X-C motif) receptor 3, *Ifnα* interferonα, *Ifnβ* interferonβ, *Actb* β-Actin, *qPCR* quantitative real-time polymerase chain reaction.
